# 血液病患者异基因造血干细胞移植后粒细胞植入前死亡原因分析

**DOI:** 10.3760/cma.j.cn121090-20231030-00240

**Published:** 2024-06

**Authors:** 竞 刘, 萌 吕, 圆圆 张, 晓冬 莫, 于谦 孙, 晨华 闫, 昱 王, 兰平 许, 晓辉 张, 开彦 刘, 晓军 黄

**Affiliations:** 北京大学人民医院，北京大学血液病研究所，国家血液系统疾病临床医学研究中心，造血干细胞移植治疗血液病北京市重点实验室，北京 100044 Peking University People's Hospital, Peking University Institute of Hematology, National Clinical Research Center for Hematological Diseases, Beijing Key Laboratory of Hematopoietic Stem Cell Transplantation for the Treatment of Hematology, Beijing 100044, China

**Keywords:** 异基因造血干细胞移植, 移植相关死亡, 死亡原因, 感染, 心脏毒性, Allogeneic stem cell transplantation, Transplant related mortality, Cause of death, Infection, Cardiac toxicity

## Abstract

**目的:**

分析异基因造血干细胞移植（allo-HSCT）患者粒细胞植入前死亡原因及人群特征。

**方法:**

对2016年1月至2023年7月在北京大学人民医院行allo-HSCT的全部7 427例患者进行回顾性分析。

**结果:**

7 427例患者中有56例（0.75％）在中性粒细胞植入前死亡，中位死亡时间为+7 d（−3 d～+38 d）。急性白血病（AL）、重型再生障碍性贫血（SAA）、骨髓增生异常综合征（MDS）患者的中位死亡时间分别为+11 d（−1 d～+38 d）、+3 d（−1 d～+ 34 d）、+16 d（−1 d～38 d）（*P*＝0.013）。主要死亡原因为感染（39.3％）、预处理心脏毒性（28.6％）和颅内出血（26.8％）。感染是引起AL和MDS患者最常见的死亡原因（55.0％、60.0％）。预处理心脏毒性所致死亡主要见于SAA患者（71.4％），而在AL患者中未见到，MDS中仅1例。53.3％的颅内出血死亡患者合并严重感染。感染、预处理心脏毒性、颅内出血引起植入前死亡的中位时间分别是+11 d（−1 d～+38 d）、+2.5 d（−1 d～+17 d）、+8 d（−3 d～+37 d）（*P*<0.001）。

**结论:**

感染是allo-HSCT患者粒细胞植入前死亡的主要原因，SAA患者应关注严重心脏毒性导致的植入前死亡。

## Abstract (en)

**Objective:**

To analyze the causes and demographic characteristics of pre-engraftment mortality in patients who underwent allogeneic hematopoietic stem cell transplantation（allo-HSCT）and investigate the risk factors and measures for preventing pre-engraftment mortality.

**Methods:**

A retrospective case analysis, involving a total of 7 427 patients who underwent allo-HSCT at Peking University People's Hospital between January 2016 and July 2023, was conducted.

**Results:**

Among the 7 427 patients who underwent allo-HSCT, 56 cases（0.75％）experienced pre-engraftment mortality. The median time to death for these 56 patients was +7（−3 to +38）days after stem cell infusion. The median times to death for patients with acute leukemia（AL）, severe aplastic anemia（SAA）, and myelodysplastic syndrome（MDS）were +11（−1 to +38）, +3（−1 to +34）, and +16（−1 to +38）days, respectively（*P*＝0.013）. The main causes of pre-engraftment mortality were infection（39.3％）, cardiac toxicity（28.6％）, and intracranial hemorrhage（26.8％）. Infection was the most common cause of pre-engraftment mortality in patients with AL and MDS（55.0％and 60.0％）, whereas cardiac toxicity was predominantly observed in patients with SAA（71.4％）, with no cases in patients with AL and only one case in patients with MDS. Among patients who died from intracranial hemorrhage, 53.3％had severe infections. The median times to death for infection, cardiac toxicity, and intracranial hemorrhage was +11（−1 to +38）, +2.5（−1 to +17）, and +8（−3 to +37）days, respectively（*P*<0.001）.

**Conclusion:**

Infection is the primary cause of pre-engraftment mortality in allo-HSCT, and severe cardiac toxicity leading to pre-engraftment mortality should be closely monitored in patients with SAA.

异基因造血干细胞移植（allo-HSCT）是恶性血液病有效乃至唯一的治愈手段，然而早期死亡仍是导致移植失败的重要原因，植入前死亡是移植后早期最为严重的事件之一[1]–[4]。现有研究大多关注移植后100 d内非复发死亡，而鲜有关于植入前死亡人群的报道。2005年欧洲血液和骨髓移植协会（EBMT）对1980至2001年间白血病移植患者的分析显示，导致早期非复发死亡的主要原因有感染、移植物抗宿主病（GVHD）等[5]。2019年EBMT更新了相关数据，显示在移植后不同时间段（超早期30 d、早期100 d、中期1年和晚期5年）死因构成比不同，感染和其他原因占移植后30 d内死亡原因的80％以上[6]。美国的一项研究显示，1997至2007年间allo-HSCT患者非复发死亡的主要原因有器官损伤、感染和严重GVHD，但未分析移植后不同时间的非复发死亡原因[7]。

近年来，预处理方案、抗感染药物、诊断技术等方面的进步使allo-HSCT的适应证扩大，适应人群扩展，接受allo-HSCT的患者数量显著增加，移植后早期死亡率及死亡原因是否随之发生变化尚不清楚。为此，我们以本中心2016年以来的allo-HSCT患者作为研究对象，分析粒细胞植入前死亡的原因和人群特征，并探讨粒细胞植入前死亡的危险因素和应对措施。

## 病例与方法

一、病例资料

本研究收集2016年1月1日至2023年7月31日在北京大学人民医院行allo-HSCT连续病例7427例。以中性粒细胞植入作为观察终点，在达到观察终点前死亡的病例作为研究对象。

二、定义

死亡原因分为感染、毒性、出血和其他。感染分为细菌、真菌、病毒、寄生虫、混合感染和未知感染[8]。中性粒细胞植入定义为连续3 d中性粒细胞绝对计数（ANC）≥0.5×10^9^/L，心脏毒性和颅内出血的定义参考文献[9]–[10]。输注造血干细胞当天定义为0 d。

三、统计学处理

应用SPSS23.0软件及R4.2进行数据分析，组间定量资料比较采用*t*检验，组间定性资料比较采用卡方检验或Fisher确切概率法，多组比较采用Kruskal-Wallis检验，双侧*P*<0.05为差异有统计学意义。

## 结果

一、植入前死亡率和患者基本特征

7 427例allo-HSCT患者中有56例（0.75％）在粒细胞植入前死亡。56例死亡患者中51例是首次移植，5例是二次移植。急性白血病（AL）20例（35.7％），骨髓增生异常综合征（MDS）10例（17.9％），重型再生障碍性贫血（SAA）21例（37.5％），其他疾病5例（8.9％）。所有患者的人口学、疾病和移植相关特征见表1。

**表1:** 56例异基因造血干细胞移植后粒细胞植入前死亡患者一般特征

患者特征	结果
性别（例，男/女）	42/14
年龄［岁，*M*（范围）］	34（1~62）
原发病［例（%）］	
急性白血病	20（33.9）
急性髓系白血病	12（21.4）
急性淋巴细胞白血病	6（10.7）
其他类型	2（3.6）
骨髓增生异常综合征	10（19.6）
LB	3（7.1）
IB	7（12.5）
再生障碍性贫血	21（37.5）
重型	16（28.6）
极重型	5（8.9）
慢性粒-单核细胞白血病	2（3.6）
慢性髓性白血病	1（1.8）
淋巴瘤	1（1.8）
遗传代谢病	1（1.8）
AL移植前疾病状态［例（%）］	
未缓解或复发	6（12.5）
缓解	14（25.0）
移植合并症指数［例（%）］	
0分	28（50.0）
1分	15（26.8）
2分	8（14.3）
5分	1（1.8）
缺失	4（7.1）
HLA不合位点［例（%）］	
0	9（16.1）
1	2（3.6）
2	4（7.1）
3	41（73.2）
供患者关系［例（%）］	
同胞	19（33.9）
父母	23（41.1）
子女	10（17.8）
非血缘	3（5.4）
旁系	1（1.8）
供患者血型［例（%）］	
相合	34（60.7）
大不合	10（17.9）
小不合	8（14.3）
双向不合	4（7.1）
预处理方案［例（%）］	
Bu+Cy±ATG	36（64.3）
Bu+Cy+Flu±ATG	12（21.4）
TBI+Cy+ATG	1（1.8）
TBI+Cy+Flu+ATG	1（1.8）
TBI+Flu±ATG	2（3.6）
Dec+Bu+Cy±ATG	2（3.6）
Flu+Cy	2（3.6）
干细胞来源［例（%）］	
骨髓	1（1.8）
外周血	17（30.4）
骨髓+外周血	38（67.8）
MNC输注量［×10^8^/kg，*M*（范围）］	9.4（5.0~14.5）
CD34^+^细胞输注量［×10^6/^kg，*M*（范围）］	2.9（0.9~10.3）

**注** LB：低原始细胞；IB：原始细胞增多；AL：急性白血病；Bu：白消安；Cy：环磷酰胺；Flu：氟达拉滨；TBI：全身放射治疗；ATG：抗胸腺细胞球蛋白；Dec：地西他滨；MNC：单个核细胞

20例AL患者中有14例（70.0％）在移植前处于缓解状态，6例（30.0％）患者是未缓解或复发状态。10例MDS患者中包括低原始细胞（LB）3例（30.0％）、原始细胞增多（IB）7例（70.0％）。SAA患者中5例（23.8％）是极重度SAA（VSAA）。发生植入前死亡患者的中位年龄为34（1～62）岁，其中≥55岁7例。SAA组患者的中位年龄低于AL组和MDS组（*P*<0.001）。MDS组全相合移植占比较高（*P*＝0.022）。1例患者造血干细胞移植合并症指数（HCT-CI）为5分，其余患者HCT-CI≤2分。AL、SAA和MDS组性别、移植年份、血型匹配、HCT-CI、干细胞来源、MNC输注量、CD34^+^细胞输注量等因素差异无统计学意义（表2）。

**表2:** 发生粒细胞植入前死亡急性白血病（AL）、再生障碍性贫血（AA）和骨髓增生异常综合征（MDS）患者临床特征比较

临床特征	AL（20例）	AA（21例）	MDS（10例）	*P*值
性别（例，男/女）	15/5	16/5	7/3	0.933
年龄［岁，*M*（范围）］	39（12~62）	23（8~55）	40（30~47）	<0.001
移植次数［例（%）］				0.138
首次	16（80.0）	20（95.2）	10（100.0）	
二次	4（20.0）	1（4.8）		
移植合并症指数［例（%）］				0.932
0分	10（50.0）	9（42.9）	6（60.0）	
1分	5（25.0）	6（28.6）	2（20.0）	
2分	3（15.0）	3（14.3）	2（20.0）	
5分	1（5.0）	0（0）		
缺失	1（5.0）	3（14.3）		
HLA不合位点［例（%）］				0.022
0	2（10.0）	2（9.5）	4（40.0）	
1	1（5.0）	1（4.8）	2（20.0）	
2	1（5.0）	0（0）	4（40.0）	
3	16（80.0）	18（85.7）		
移植年份［例（%）］				0.257
2016年	3（15.0）	2（9.5）	0（0）	
2017年	2（10.0）	3（14.3）	4（40.0）	
2018年	0（0）	5（23.8）	1（10.0）	
2019年	3（15.0）	3（14.3）	1（10.0）	
2020年	0（0）	3（14.3）	0（0）	
2021年	3（15.0）	1（4.8）	1（10.0）	
2022年	7（35.0）	7（14.3）	1（10.0）	
2023年	2（10.0）	1（4.8）	0（0）	
供患者关系［例（%）］				0.055
同胞	5（25.0）	6（28.6）	6（60.0）	
父母	6（30.0）	13（61.9）	3（30.0）	
子女	7（35.0）	0（0）	1（10.0）	
非血缘	1（5.0）	2（9.5）	0（0）	
旁系	1（5.0）	0（0）	0（0）	
供患者血型［例（%）］				
相合	11（55.0）	13（61.9）	6（60.0）	0.976
大不合	4（20.0）	3（14.3）	2（20.0）	
小不合	5（25.0）	3（14.3）	2（20.0）	
双向不合	0（0）	2（9.5）	0（0）	
预处理方案［例（%）］				
BuCy±ATG	11（57.9）	15（71.4）	6（60.0）	0.373
BuCy+Flu±ATG	2（10.5）	6（28.6）	2（20.0）	
TBI+Cy+ATG	1（5.3）	0（0）	0（0）	
TBI+Cy+Flu+ATG	1（5.3）	0（0）	0（0）	
TBI+Flu±ATG	0（0）	0（0）	2（20.0）	
Dec+BuCy±ATG	2（10.5）	0（0）	0（0）	
Flu+Cy	2（10.5）	0（0）	0（0）	
干细胞来源［例（%）］				0.982
骨髓	0（0）	1（4.8）	0（0）	
外周血	12（57.9）	1（4.8）	4（40.0）	
骨髓+外周血	7（36.8）	19（90.5）	6（60.0）	
MNC输注量［×10^8^/kg，*M*（范围）］	9.9（6.4~14.5）	8.7（4.9~14.3）	8.1（5.7~14.0）	0.485
CD34^+^细胞输注量［×10^6^/kg，*M*（范围）］	2.4（1.2~8.6）	3.7（0.9~10.3）	2.1（1.2~5.5）	0.245

**注** HLA：人类白细胞抗原；Bu：白消安；Cy：环磷酰胺；Flu：氟达拉滨；TBI：全身放射治疗；ATG：抗胸腺细胞球蛋白；Dec：地西他滨；MNC：单个核细胞

二、死亡时间

56例患者死亡的中位时间为+7 d（−3 d～+38 d），移植前死亡4例（7.1％），移植当天死亡2例（3.6％），移植后死亡50例（89.3％）。AL及非霍奇金淋巴瘤（NHL）、SAA及遗传病（IEM）、MDS/骨髓增殖性肿瘤（MPN）组的死亡中位时间分别为+11 d（−1 d～+38 d）、+3 d（−1 d～+34 d）、+16 d（−1 d～+38 d）（*P*＝0.013）。死因为感染、心脏毒性、颅内出血和其他患者的中位死亡时间分别为+11 d（−1 d～+38 d）、+2.5 d（−1 d～+17 d）、+8 d（−3 d～+37 d）、+18 d（+6 d～+34 d）（*P*<0.001）。

三、死亡原因

死亡原因依次为感染（39.3％，22/56）、预处理心脏毒性（28.6％，16/56）、颅内出血（26.8％，15/56）、其他（5.4％，3/56，移植相关血栓性微血管所致多脏器功能衰竭、横纹肌溶解导致的多脏器功能衰竭、呼吸衰竭各1例），详见表3。

**表3:** 不同疾病亚组患者死亡原因分析［例（％）］

组别	例数	感染				预处理心脏毒性	脑出血	其他
		细菌	真菌	混合	不明			
急性白血病	20	7（35.0）	1（5.0）	2（10.0）	1（5.0）	0（0）	8（40.0）	1（5.0）
再生障碍性贫血	21	2（4.8）	1（4.8）	0（0）	0（0）	15（71.4）	2（9.5）	1（4.8）
骨髓增生异常综合征	10	3（30.0）	0（0）	1（10.0）	2（20.0）	1（10.0）	3（30.0）	0（0）

四、疾病类型与死亡原因

在AL组，感染和颅内出血是导致死亡的常见原因，分别占55.0％、40.0％，未发生预处理心脏毒性相关死亡。在MDS组，感染、颅内出血、预处理心脏毒性分别占死因的60.0％、30.0％、10.0％。而在SAA组，最常见的死亡原因是预处理心脏毒性（71.4％），其次是感染（14.3％）和颅内出血（9.5％）。详见表2。

五、感染

感染是导致22例（39.3％）患者死亡的主要原因，在颅内出血为主要死因的15例患者中有8例在颅内出血时合并有感染。感染在全部患者及AL组和MDS组中是排在首位的死亡原因。感染导致死亡的中位时间是+11 d（−1 d～+38 d）。

1. 感染部位：28例感染患者中有23例（82.1％）有血流感染，其他常见的感染部位还有肺（5例，17.6％）、中枢神经系统（2例，7.1％）和软组织（1例，3.6％）。

2. 病原体：根据导致感染的病原体不同将感染分为细菌、真菌、病毒、寄生虫、混合和未知感染。细菌感染（67.9％，19/28）最常见，其次是混合（10.3％，3/28）感染和真菌感染（7.1％，2/28），其余4例（14.3％）为未知感染。3例混合感染中细菌、真菌混合感染2例，病毒混合感染1例。有23例患者从血液、痰液及其他分泌物或体液标本中培养出病原体，1例通过二代测序在无菌体液标本中检出病原体特异性序列。共检出革兰阴性杆菌21例、革兰阳性菌7例、念珠菌2例、阿萨希毛孢子菌1例、微小根毛霉1例。革兰阴性杆菌中最常见的是耐碳青霉烯类肠杆菌（11例）、产超广谱β-内酰胺酶肠杆菌（6例）、多重耐药鲍曼不动杆菌（3例）。革兰阳性菌包括耐甲氧西林葡萄球菌（2例）和肠球菌（1例）、耐万古霉素肠球菌（2例）和其他肠球菌（2例）。2例念珠菌分别是白色念珠菌和热带念珠菌各1例。

3. 细菌定植：56例死亡患者中有47例在进入层流室第1天进行了口咽、鼻、腋下和肛周拭子细菌培养。47例患者中有12例（25.5％）患者检出细菌定植：口咽拭子6例，鼻拭子4例，肛拭子2例。其中4例（8.5％）定植菌是耐碳青霉烯类肠杆菌，其他定植菌包括铜绿假单胞菌2例、鲍曼不动杆菌2例和葡萄球菌2例。其中7例患者导致感染的致病菌与定植菌一致。在主要死亡原因为感染、预处理毒性和脑出血患者中入层流室时细菌定植检出率分别为31.8％（7/22）、12.5％（2/16）和20.0％（3/15）（*P*＝0.170）。

六、预处理相关心脏毒性

16例（28.6％）患者死于预处理导致的心脏毒性，仅次于感染。死于心脏毒性的16例患者中有15例SAA患者和1例输血依赖的MDS-LB患者，其中4例是VSAA，而在AL患者中未发生心脏毒性相关死亡。心脏毒性相关死亡发生的中位时间是+2.5 d（−1 d ～ +17 d），早于感染和脑出血导致的死亡时间。

七、颅内出血

15例（26.8％）患者死于颅内出血。颅内出血导致的死亡更常见于AL和MDS患者，在AL、MDS、SAA患者中的占比分别为40.0％、30.0％、9.5％。颅内出血导致死亡的中位时间为+8 d（−3 d～+37 d）。有8例（53.3％）颅内出血死亡患者在颅内出血发生时合并严重感染，3例（20.0％）患者合并血小板无效输注，其中1例抗血小板抗体阳性，2例供者特异性抗体（DSA）抗体阳性。颅内出血发生当天中位血小板计数为6（1～34）×10^9^/L，尽管9例血小板计数低于10×10^9^/L的患者均输注了血小板，仍未能阻断颅内出血发生。2例患者既往有高血压病史，移植前血压控制在正常范围。

## 讨论

植入前死亡是移植早期最为严重的事件之一，但鲜有关于植入前死亡率和人群特征的报道。我们总结了迄今为止最大数量的allo-HSCT患者植入前死亡患者数据集，结果显示allo-HSCT植入前死亡率为0.75％。文献检索未见分析allo-HSCT患者植入前死亡率的大宗病例报道。2021年EBMT工作组报道，1980至2001年、2002至2015年allo-HSCT后超早期（≤30 d）死亡率分别为6.13％（1 905/31 128）、4.14％（2 654/64 661）（*P*<0.001）[6]。2021年韩国报道国家2003至2015年AL患者allo-HSCT后50 d内移植相关死亡率（TRM）为2.9％，2003至2009年、2010至2015年间分别是2.4％、3.0％（*P*＝0.210）[11]。本研究中allo-HSCT患者粒细胞植入前死亡的中位时间为+7 d（−3 d～+38 d），本组病例移植年份迟于上述报道，疾病类型纳入了AL、SAA、MDS、淋巴瘤、骨髓增殖性疾病和遗传代谢病等，而上述报道仅纳入了白血病患者。由于时间、人群、疾病和移植方案存在差异，在不同研究之间直接比较早期死亡率是不合适的。本研究中allo-HSCT患者植入前死亡率较低，显示我国allo-HSCT安全性较高，并说明植入前死亡是移植后超早期死亡的重要原因。

在本组病例中，感染是引起植入前死亡最常见的原因（占39.3％），而在AL和MDS患者的死亡原因中占比达到50％以上。这与EBMT报道感染占白血病患者allo-HSCT后30 d内死亡原因的54.3％（1 442/2 654）[6]、Kong等[11]报道感染占AL患者allo-HSCT后50 d内死亡原因66.9％的研究结果一致。Moghnieh等[12]研究认为SAA是植入前发生菌血症的独立危险因素，但在本研究中感染引起的植入前死亡在AL和MDS患者中占比显著高于SAA患者，这可能是由于在SAA患者中预处理心脏毒性是导致死亡的首要原因且其发生时间显著早于感染所致死亡。

本组病例最常见的感染部位是血流感染，其次是肺和中枢神经系统，与文献[12]–[14]报道的植入前感染特征一致。革兰阴性杆菌，尤其耐碳青霉烯类肠杆菌和产超广谱β-内酰胺酶肠杆菌是导致本组病例植入前感染死亡的主要病原体，对于出现此类病原体感染的患者应高度警惕死亡风险。既往研究显示，影响植入前感染发生的高危因素有老年、疾病未缓解、HLA不合、无关供者、脐带血移植、HCI-CI高分、植入失败和肠道菌群特征等[14]–[18]。而本研究植入前死亡患者中老年、疾病未缓解状态、HCT-CI≥ 3分的患者占比并不高，发生死亡的中位时间也早于文献报道的中性粒细胞植入时间，不同疾病亚组间人群特征除了SAA患者年龄低于AL和MDS患者外，其他因素差异无统计学意义。本研究未进行肠道菌群的检测，但大部分患者在入层流室当天进行了口咽、鼻腔、腋下和肛拭子定植细菌筛查，结果显示在植入前死亡患者中耐碳青霉烯类肠杆菌定植率与既往文献报道接近，超过半数的患者定植细菌与导致感染的致病菌的菌种相同，提示筛查定植细菌可能有助于感染防治[19]。

本组病例中预处理心脏毒性导致的植入前死亡主要发生在SAA患者，在AL中未见到心脏毒性导致的植入前死亡，在MDS-LB中有1例，提示现有的移植前心脏风险评估在SAA和MDS-LB患者中可能不够。本中心Xu等[10]报道单倍体移植SAA患者中Ⅲ/Ⅳ级心脏毒性的发生率为5.6％，发生严重心脏毒性患者的生存率显著低于无严重心脏毒性患者（12.5％对89.6％，*P*<0.001），是影响SAA患者单倍体移植预后的独立危险因素。Duléry等[20]报道，在后置环磷酰胺（PT-Cy）方案allo-HSCT恶性血液病患者中早期心脏事件发生率19％，其中严重心脏事件为直接死因的发生率为3/136（2.2％）。一项美国单中心20年的移植数据显示致命心脏毒性时间发生率<1％[21]。由于人群、预处理方案等不同，直接在不同研究间比较心脏毒性是不合适的。我中心曾报道SAA单倍体移植发生严重心脏毒性的危险因素有移植前美国东部肿瘤协作组（ECOG）评分≥2分、12导联心电图ST-T段异常、高甘油三酯血症和环磷酰胺剂量≥ 1.8 g·m^−2^·d^−1^，具有心脏毒性高危因素的SAA患者采用减低剂量环磷酰胺和氟达拉滨为基础的预处理方案可降低严重心脏毒性的发生率和死亡率[10],[22]。因此建议SAA患者allo-HSCT前应充分评估预处理心脏毒性风险，对有心脏毒性高危因素的患者进行减低毒性方案预处理可能有助于减少心脏毒性导致的死亡。

颅内出血是allo-HSCT患者少见但严重的合并症[23]–[25]。本研究发现有53.3％（8例）的颅内出血死亡患者合并严重感染，3例血小板输注无效，提示感染仍是导致颅内出血死亡的重要诱因。

许多研究报道了随着时间推移移植后早期死亡率与早年间相比显著下降，并将生存改善的主要原因归属于预处理方案改进、抗感染策略的进步和优化供者选择等[6],[11],[26]。结合本研究显示的植入前死亡原因和人群特征，积极防治感染是减少植入前死亡的重要措施，对于SAA患者，通过风险预测模型识别严重心脏毒性高危患者，并优化预处理方案，是可能降低植入前死亡的方法。本研究是一项单中心回顾性研究，植入前死亡发生率低导致病例数少。

## References

[1] Chang YJ, Pei XY, Huang XJ (2022). Haematopoietic stem-cell transplantation in China in the era of targeted therapies: current advances, challenges, and future directions[J]. Lancet Haematol.

[2] Zhang XH, Chen J, Han MZ (2021). The consensus from The Chinese Society of Hematology on indications, conditioning regimens and donor selection for allogeneic hematopoietic stem cell transplantation: 2021 update[J]. J Hematol Oncol.

[3] Passweg JR, Baldomero H, Chabannon C (2021). Hematopoietic cell transplantation and cellular therapy survey of the EBMT: monitoring of activities and trends over 30 years[J]. Bone Marrow Transplant.

[4] D'Souza A, Fretham C, Lee SJ (2020). Current use of and trends in hematopoietic cell transplantation in the United States[J]. Biol Blood Marrow Transplant.

[5] Gratwohl A, Brand R, Frassoni F (2005). Cause of death after allogeneic haematopoietic stem cell transplantation (HSCT) in early leukaemias: an EBMT analysis of lethal infectious complications and changes over calendar time[J]. Bone Marrow Transplant.

[6] Styczyński J, Tridello G, Koster L (2020). Death after hematopoietic stem cell transplantation: changes over calendar year time, infections and associated factors[J]. Bone Marrow Transplant.

[7] Gooley TA, Chien JW, Pergam SA (2010). Reduced mortality after allogeneic hematopoietic-cell transplantation[J]. N Engl J Med.

[8] Hahn T, Sucheston-Campbell LE, Preus L (2015). Establishment of definitions and review process for consistent adjudication of cause-specific mortality after allogeneic unrelated-donor hematopoietic cell transplantation[J]. Biol Blood Marrow Transplant.

[9] Ren X, Huang Q, Qu Q (2021). Predicting mortality from intracranial hemorrhage in patients who undergo allogeneic hematopoietic stem cell transplantation[J]. Blood Adv.

[10] Xu ZL, Xu LP, Zhang YY (2019). Incidence and predictors of severe cardiotoxicity in patients with severe aplastic anaemia after haploidentical haematopoietic stem cell transplantation [J]. Bone Marrow Transplant.

[11] Kong SG, Jeong S, Lee S (2021). Early transplantation-related mortality after allogeneic hematopoietic cell transplantation in patients with acute leukemia[J]. BMC Cancer.

[12] Moghnieh R, Tamim H, Abyad A (2020). Pre-engraftment infectious complications and patient outcomes after allogeneic hematopoietic cell transplantation: a single-center experience from Lebanon[J]. Infection.

[13] Girmenia C, Bertaina A, Piciocchi A (2017). Incidence, risk factors and outcome of pre-engraftment gram-negative bacteremia after allogeneic and autologous hematopoietic stem cell transplantation: an italian prospective multicenter survey[J]. Clin Infect Dis.

[14] Blennow O, Ljungman P, Sparrelid E (2014). Incidence, risk factors, and outcome of bloodstream infections during the pre-engraftment phase in 521 allogeneic hematopoietic stem cell transplantations[J]. Transpl Infect Dis.

[15] Mikulska M, Raiola AM, Galaverna F (2018). Pre-engraftment bloodstream infections after allogeneic hematopoietic cell transplantation: impact of T cell-replete transplantation from a haploidentical donor[J]. Biol Blood Marrow Transplant.

[16] Harris B, Morjaria SM, Littmann ER (2016). Gut microbiota predict pulmonary infiltrates after allogeneic hematopoietic cell transplantation[J]. Am J Respir Crit Care Med.

[17] Kikuchi M, Akahoshi Y, Nakano H (2015). Risk factors for pre- and post-engraftment bloodstream infections after allogeneic hematopoietic stem cell transplantation[J]. Transpl Infect Dis.

[18] Gudiol C, Garcia-Vidal C, Arnan M (2014). Etiology, clinical features and outcomes of pre-engraftment and post-engraftment bloodstream infection in hematopoietic SCT recipients[J]. Bone Marrow Transplant.

[19] Yan L, Sun J, Xu X (2020). Epidemiology and risk factors of rectal colonization of carbapenemase-producing Enterobacteriaceae among high-risk patients from ICU and HSCT wards in a university hospital[J]. Antimicrob Resist Infect Control.

[20] Duléry R, Mohty R, Labopin M (2021). Early cardiac toxicity associated with post-transplant cyclophosphamide in allogeneic stem cell transplantation[J]. JACC CardioOncol.

[21] Murdych T, Weisdorf DJ (2001). Serious cardiac complications during bone marrow transplantation at the University of Minnesota, 1977-1997[J]. Bone Marrow Transplant.

[22] Lin F, Zhang Y, Han T (2022). A modified conditioning regimen based on low-dose cyclophosphamide and fludarabine for haploidentical hematopoietic stem cell transplant in severe aplastic anemia patients at risk of severe cardiotoxicity[J]. Clin Transplant.

[23] Cai X, Fu HX, Mo XD (2020). Comparison of hemorrhagic and ischemic stroke after allogeneic hematopoietic stem cell transplantation[J]. Bone Marrow Transplant.

[24] Zhang XH, Wang QM, Chen H (2016). Clinical characteristics and risk factors of Intracranial hemorrhage in patients following allogeneic hematopoietic stem cell transplantation[J]. Ann Hematol.

[25] Najima Y, Ohashi K, Miyazawa M (2009). Intracranial hemorrhage following allogeneic hematopoietic stem cell transplantation[J]. Am J Hematol.

[26] Tanaka Y, Kurosawa S, Tajima K (2016). Analysis of non-relapse mortality and causes of death over 15 years following allogeneic hematopoietic stem cell transplantation[J]. Bone Marrow Transplant.

